# Mechanisms of DNA Damage Response to Targeted Irradiation in Organotypic 3D Skin Cultures

**DOI:** 10.1371/journal.pone.0086092

**Published:** 2014-02-05

**Authors:** Anna Acheva, Mihaela Ghita, Gaurang Patel, Kevin M. Prise, Giuseppe Schettino

**Affiliations:** 1 Centre for Cancer Research and Cell Biology, Queen's University Belfast, Belfast, United Kingdom; 2 Environmental Radiation Surveillance, Radiation and Nuclear Safety Authority, Helsinki, Finland; 3 National Physical Laboratory, Teddington, London, United Kingdom; University of California Davis, United States of America

## Abstract

DNA damage (caused by direct cellular exposure and bystander signaling) and the complex pathways involved in its repair are critical events underpinning cellular and tissue response following radiation exposures. There are limited data addressing the dynamics of DNA damage induction and repair in the skin particularly in areas not directly exposed. Here we investigate the mechanisms regulating DNA damage, repair, intracellular signalling and their impact on premature differentiation and development of inflammatory-like response in the irradiated and surrounding areas of a 3D organotypic skin model. Following localized low-LET irradiation (225 kVp X-rays), low levels of 53BP1 foci were observed in the 3D model (3.8±0.28 foci/Gy/cell) with foci persisting and increasing in size up to 48 h post irradiation. In contrast, in cell monolayers 14.2±0.6 foci/Gy/cell and biphasic repair kinetics with repair completed before 24 h was observed. These differences are linked to differences in cellular status with variable level of p21 driving apoptotic signalling in 2D and accelerated differentiation in both the directly irradiated and bystander areas of the 3D model. The signalling pathways utilized by irradiated keratinocytes to induce DNA damage in non-exposed areas of the skin involved the NF-κB transcription factor and its downstream target COX-2.

## Introduction

Ionizing radiation is a genotoxic agent producing wide range of DNA alterations (i.e. strand breaks, base damages and cross-links) which after processing through the cellular repair machinery determine the variety and severity of cellular and tissue effects. Double strand break (DSB) is the critical lesion which can lead to cell death as well as genomic instability if unrepaired or miss-repaired. In eukaryotic cells, DSBs are repaired via two main repair pathways, the non-homologous end joining (NHEJ) and the homologous recombination (HR). These processes have been extensively studied and the main proteins involved identified and characterized. For NHEJ, DNA-dependent protein kinases (Ku70 and Ku80) and DNA ligase XRCC4 [1]–[2] have been identified as key components whilst for the HR, the MRN complex and Rad52/Rad54 appear to play crucial roles [3]. In general, proteins involved in DNA repair processes are classified as damage sensors, signal transducers and effectors. Among the sensors, ATM, ATR and DNA-PK play a central role as they are activated at an early stage and responsible for an efficient triggering of repair mechanisms. One of the important steps in evaluation the damage severity and cellular ability to advance through the cell cycle is activation of p21^WAF1/Cip1^. p21 inhibits the cell cycle dependent kinases (CDK) via supressing Cyclin E and Cyclin A-associated CDK2-activities, thus blocking the cell cycle progression [4]. It acts as cell cycle checkpoint and is able to block the cell cycle in both G1/S and G2/M phases. It has also been pointed as one of the main factors inducing p53-dependent apoptosis [4]–[5]. p21 was reported to be up-regulated in the initial phases of human primary keratinocyte terminal differentiation and to be decreasing at the late stages of the process [5]. The protein increase has also been suggested as necessary step in the removal of cells with accumulated DNA damage via apoptosis. When sub-lethal DNA damages are induced, p21 acts as an inducer of cell cycle arrest and facilitates damage repair [4]–[6].

DNA lesions and the efficiency of the repair processes are also responsible for the activation and modulation of proteins and enzymes such as NF-kB, COX-2, iNOS [7] which directly regulate the tissue inflammatory reactions.

Our current knowledge of the proteins and mechanisms involved in the repair of DNA DSBs is mainly based on the response of 2D cell cultures irradiated with conventional broad fields of high doses of low and high LET radiation. Despite the acknowledged importance of inter- and intra-cellular signaling and the influence of the tissue microenvironment on the individual cellular response, there is limited information regarding the DNA repair processes in 3D systems especially under non-uniform exposure scenarios such as those occurring naturally or in clinical practice. This is a fundamental requirement to further understand the complex machinery of DNA repair, estimate human health risks to radiation exposures and improve current clinical protocols employing ionizing radiation.

Use of 3D organotypic cultures including co-culturing of different cell types is now a well-established technique and several models have been very successful employed in epidermal biology [8]–[9]. Skin models, in particular, have been established and characterized by co-culturing keratinocytes on fibroblast embedded collagen gels [8]–[11]. These cultures have the typical differentiation pattern and functional features of *in vivo* epidermis, including stratification into basal, spinous, granular and cornified layers [11]. They therefore represent an optimal system to perform mechanistic studies to evaluate the damaging and repair kinetics of ionizing radiation in a 3D architecture.

53BP1 is a human BRCT protein which binds to DNA DSB (double strand brakes) areas. Following DNA damage, 53BP1 is phosphorylated and can be visualized as foci at the DSB sites via immunofluorescence technique [12]. The 53BP1 foci induced in epidermal cells followed a dose-dependent induction and biphasic decrease with time post irradiation. There has also been reported persistent foci growth up to a few hours which was suggested to be connected with G1 arrest and especially with amplifying the G1 checkpoints signalling sufficiently to induce p53 phosphorylation in cells with very low number of remaining 53BP1 foci and activate p21-induced cell cycle arrest. This process has been regarded as a tumor suppressor mechanism preventing cells with remaining DNA damage from division [13].

Existing 3D data are mainly related to DSB induction, micronuclei formation and apoptosis in the directly irradiated cells with some studies of bystander effects limited to high LET radiation, i.e. proton and alpha particle beams [14]–[17]. Using particle irradiation, DNA damage measurements in organotypic models have shown γ-H2AX foci induction in bystander cells up to 2.5 mm away from the irradiated area. Interestingly, the formation of the foci in the bystander cells has delayed kinetics and reaches a maximum at 12–48 h after irradiation [14], [17] compared to the 30 min – 1 h peak in the 2D cultures. They also reported increased levels of micronuclei, senescence and apoptosis with premature differentiation up to 9 mm from the irradiated sites. The observed apoptotic and senescence response in cells surrounding irradiated areas was described as a local protective response to potentially carcinogenic cellular changes [14]. With regards to low LET radiation, Suzuki et al. [18] showed induction of 53BP1 foci in the basal and partially in the spinous layer of a 3D skin model (i.e. rapidly dividing and undifferentiated cells) following X-irradiation. The foci followed a dose-dependent induction and biphasic decrease with time post irradiation although with significant differences from the equivalent 2D cultures which was attributed to dosimetry and foci quantification. There have also been reported persistent foci growth up to a few hours which was suggested to be connected with G1 arrest and amplification of G1 checkpoint signaling sufficient to induce p53 phosphorylation in cells with very low number of residual damage. This process has been regarded as protective mechanism where cells with DNA damage are prevented from division [13]. On the other hand, evidences that foci growth is connected with chromatin remodelling in order to facilitate DNA repair have also been reported in 2D cultures [18]–[20].

Both the yields and quality of radiation-induced DNA damage as well as the way such damage is processed by cells also determines the skin response towards an inflammatory reaction. In the early stages of inflammation, pro-inflammatory cytokine production is triggered and it continues as a cascade during the process of the development of a cutaneous reaction [21]. The inflammatory skin reactions following radiotherapy have been suggested to be driven by the transcription factor NF-κB and its downstream targets COX-2 and iNOS [7]. NF-κB proteins form homo- and heterodimers which are activated by different external stimuli such as DNA DSB [7], [22]. When activated from various pro-inflammatory cytokines, NF-κB triggers the expression of genes responsible for cellular proliferation, anti-apoptotic genes and also has an up-regulatory role in angiogenesis. The disrupted regulation of this transcription factor is therefore thought to be involved in various chronic inflammatory diseases including skin reactions, cancer formation and also in resistance to apoptosis-inducing cancer treatment [22]. The COX converts of arachidonic acid to prostanoids - secondary pro-inflammatory signalling molecules. It has two iso-forms COX-1 which is constitutively expressed in skin and COX-2 which is the inducible form produced after stimulation with cytokines and mitogens [23]. COX-2 has been indicated to be involved in inflammation as it has been shown to be up-regulated in allergic asthma, rheumatoid arthritis, lipopolysaccharide- and TPA-induced skin inflammation, UVA and UVB and radiation-induced erythema [23]–[25].

The present work, in a 3D skin model, investigates the mechanisms that interlink the initial radiation-induced DNA damage, its repair, the intracellular signaling and the subsequent cellular effects such as hyperproliferation, increased rate of terminal differentiation and changes in the expression of differentiation markers. Furthermore, we studied the signaling pathways that cells utilize to transmit pro-inflammatory signals and to exert radiation-induced skin effects within the model. The study focused on both directly exposed and neighboring cells using low LET radiation as conventionally employed for radiotherapy and diagnostic applications. Numerous studies on direct and bystander effects in 2D cell cultures have been used to extrapolate the DNA damaging effect of ionizing radiation in 3D models. However, more recent studies [13]–[14], [17]–[20], showed substantial differences in the DNA damage response between 2D and 3D models. This clearly indicates that direct and non-targeted effects of radiation should be evaluated together and in a model that structurally more closely relates to normal tissue in order to be included in the prognostic features of the response in the radiotherapy patients [7], [21]–[26]. Comparison with the corresponding 2D models could also provide valuable information regarding the effect of cell organization on DNA repair pathways. A novel low LET microcollimator (1–10 µm wide stripes of 30 KVp X-rays) represents a unique approach to study localized effects of low LET irradiation in 3D models. The 3D data reported to date are mainly based on high LET radiation and need validation with more clinically relevant irradiation scenarios. Finally, our data indicate that differences in cellular activities may alter the DNA repair processes promoting apoptosis in the 2D model and premature differentiation in 3D systems.

## Materials and Methods

### Cell culture

N/TERT-1 keratinocytes immortalized by transfection to express TERT [27]–[28] obtained from Dr Rheinwald from Harvard Institutes of Medicine, Boston, MA, were grown in Keratinocyte Serum Free media (K-sfm) (Invitrogen, Carlsbad, CA, USA). The media has been modified as suggested by the supplier and supplemented with penicillin/streptomycin. The subculturing of the cells was done by trypsinization with Trypsin/EDTA (GIBCO, Invitrogen, Carlsbad, CA). The trypsin was neutralized with equal volume of DMEM (PAA, Pasching, Austria) medium containing 10% Fetal Calf Serum (FCS, PAA, Pasching, Austria). Cells were pelleted by low speed centrifugation (∼400 g) for 5 minutes and re-suspended in GIBCO K-SFM medium.

### Organotypic raft cultures

N/TERT-1 keratinocytes were expanded to 60–70% confluence in T75 flasks. J2-3T3 fibroblasts were routinely cultured in DMEM supplemented with 10% FCS (PAA, Pashing, Austria). J2 cells were treated with Mitomycin C (Sigma-Aldrich, St. Louise, MO, USA) (4 µg/ml) to block the mitosis for minimum 2 h before using them for raft cultures. Fibroblasts were then trypsinised, centrifuged and added to keratinocytes in T25 flasks (∼1∶3 fibroblasts∶keratinocytes ratio) J2-3T3 were added to keratinocytes in E-media (formulation described in [29]), containing 10 ng/ml EGF (Calbiochem, La Jolla, CA, USA) and co-cultured overnight. Collagen Type I plugs containing J2-3T3 were prepared from 3 mg/ml final concentration Rat tail collagen (acidic) (BD, Bedford, MA, USA), 5×DMEM (MP Biomedicals, Illkirch, France), 1M NaOH filter sterilized to a final volume of 2 ml per plug. The collagen gels with added 4.5×10^5^ J2 3T3 cells were let to solidify in hanging membrane inserts with 6-well companion plates (BD Falcon, NJ, USA). Once the gels have polymerized, 1×10^6^ N/TERT-1 keratinocytes per plug were plated on the gels in membrane inserts and allowed to attach for 1 h. The cultures were fed with E-media with EGF. On the next day, media from the top chamber was aspirated and cultures were fed from the bottom chamber with E-media without EGF as the collagen gels were exposed to the air-liquid interface to stimulate differentiation. The 3D skin cultures were fed daily for the first 2–3 days then every 2 days. The cultures were harvested at day 11, fixed in 4% paraformaldehyde and processed for paraffin embedding, sectioning for immunofluorescence and H&E staining.

### Irradiation and dosimetry

**Figure 1:**
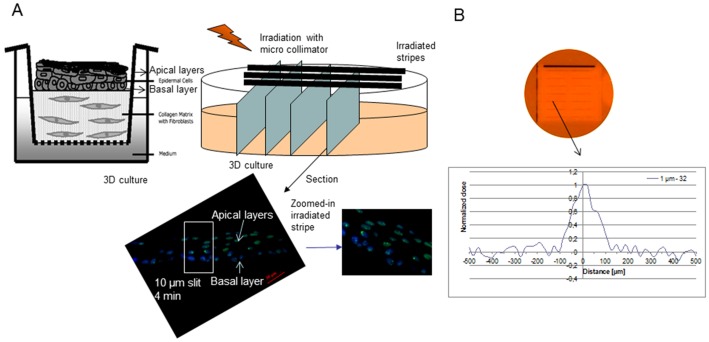
Schematic representation of the irradiation set-up. Schematic representation of the microcollimator irradiation and DNA damage foci on 3D skin cultures (A). The cultures were either half shielded or exposed to microcollimated low LET irradiation from the apical side. For DNA damage foci cultures were 4% paraformaldehyde fixed and paraffin embedded, then sectioned as on (A) for immunofluorescence. For western blot experiments, cultures were cut in halves and lysed immediately as described in Materials and methods. On the bottom panels stained for 53 BP1 sections (green, 53BP1, blue – DAPI nuclear staining). Dose profile of the lines of irradiation as detected on gafchromic film after 30 kVp, 45 mA, 1 µm microcollimator exposure (B). Scale bar 20 µm.

Radiation exposures were performed using a 225 KVp X-ray source (XRAD 225 from Precision X-rays Inc. N. Branford, CT, USA) at dose rate of 0.591 Gy/min measured with a Farmer ionization chamber (PTW, Freiburg, Germany). The irradiation experiments with 50% shielding of the 3D cultures were performed using a custom designed frame and two 2 cm thick MCP-96 lead-containing alloy blocks. The efficiency of shielding was confirmed by Gafchromic film measurement with less than 2.3% of the dose delivered reaching under the shielded area and a sharp transition (dose falls from 90% to 10% within 2 mm). Additionally we used gallium arsenide microcollimator from MicroLeman (Lausanne, Switzerland) [30], which allows exposure of samples with localized (1–10 µm wide stripes) oft 30 KVp X-rays (Figure 1A).

The collimator dose rate was ∼0.014 Gy/min at 30 kVp, 45 mA. The 3D cultures were exposed to a dose of 0.095 Gy and the irradiation lines were monitored on a gafchromic film placed underneath the cultures (Figure 1B). Samples were fixed 30 min later. The 3D cultures were then cut into stripes at 90° angle relatively to the plane of irradiation (monitored on the gafchromic film). This allowed the whole width of the irradiated stripes and the corresponding DNA damage induced to be scored across the full tissue thickness.

### Immunofluoresce staining of 2D cultures

N/TERT-1 cells grown in 500 µm grid μ-dishes 35 mm (Ibidi, Martinsried, Germany) or on 16 mm diameter coverslips were fixed for 10 min with 1∶1 acetone∶methanol at −20°C, washed with PBS and incubated with 0.5% Triton X-100 solution (w/v) in PBS buffer for 20 min. The cells were treated with blocking solution (0.2% non-fat milk, 0.1% Triton X-100, 5% FCS) for 1 h. This was followed by incubation with 1∶10000 diluted 53BP1 primary rabbit antibody (Novus Biologicals, Littleton, CO, USA) for 1 h at room temperature and 1∶1000 diluted goat anti-rabbit Alexa 488 conjugated antibody (Invitrogen, Carlsbad, CA, USA) for 1 h at 4°C in the dark. The nuclei were counterstained with DAPI (Invitrogen, Carlsbad, CA, USA) and mounted on microscopic slides with anti-fading media. 53BP1 foci were scored by eye in >50 cells per sample under inverted microscope Zeiss Axiovert 200 M (Carl Zeiss, Göttingen, Germany). Pictures were taken with digital camera Axio Cam MRm (Carl Zeiss, Göttingen, Germany) and the 53BP1 foci size (i.e. Full Width Half Maximum, FWHM) measured with Image J 1.04 software (NIH, Bethesda, MA, USA).

### Immunofluoresce staining of 3D organotypic skin cultures

Culture sections were de-paraffinized with xylene and decreasing alcohol concentrations. Following that, sections were subjected to antigen unmasking where required (i.e. 53BP1 and Filaggrin) with citrate buffer (20 min boiling, followed by 20 min on the bench). The slides were blocked in 10% FCS in 0.2% PBS-TritonX-100 for 30 min and incubated with primary antibodies in the following dilutions: 1∶50 for Cytokeratin 1 (Vector Laboratories, Burlingame, CA, USA); 1∶100 for Filaggrin (AnaSpec, San Jose, CA, USA) and 1∶200 for 53BP1 (Novus Biologicals, Novus Biologicals, Littleton, CO, USA). After overnight incubation with the primary antibody or 1 h for 53BP1 at room temperature, the sections were washed with 0.1% Triton X-100 in PBS and incubated with secondary Alexa 488 or 568 conjugated antiboby (Molecular probes, Invitrogen, OR, USA) for 1 h at room temperature. After washing with washing buffer slides were counterstained with DAPI containing mounting media Vectashield (Vector Laboratories, Burlingame, CA, USA) and sealed with clean nail varnish.

### Western blotting

N/TERT-1 grown in 2D or differentiated in 3D organotypic cultures (total cultures) were lysed with Lysis Buffer containing 50 mM Tris, HCl pH 8.0, 150 mM NaCl, 1% Triton X-100 and protease and phosphatase inhibitor cocktail (Roche, Mannheim, Germany). The protein concentration was measured according the Bradford method (BioRad, Munich, Germany). The protein solution was diluted in NuPage loading buffer (Invitrogen, Carlsbad, CA, USA) and 30 or 60 µg per line loaded on 4–12% Bis-Tris NuPage pre-casted gels (Invitrogen, Carlsbad, CA, USA). The separated proteins were transferred on nitrocellulose membrane using iBlot semi-dry transfer apparatus. Membranes were blocked with blocking buffer (5% skimmed milk, 0.1% Tween 20 in PBS) for 1 h at room temperature and incubated with primary antibody against p21 (Upstate, Temecula, CA), COX-2 (Millipore, Temecula, CA) 1∶500; Phospho-p38Tyr^180/182^ (Cell Signaling, Denver, MA, USA) 1∶1000; GAPDH and β-actin 1∶5000 (Sigma-Aldrich, St. Louise, MO, USA) overnight at 4°C. The membranes were washed with 0.1% PBS-T and incubated with secondary HRP- conjugated antibody (ECL™ Anti-Mouse/Anti Rabbit IgG, GE Healthcare, Little Chalfont, UK) for 1 h at room temperature. After washing with 0.1% PBS-T membranes were incubated with SuperSignal ECL (Thermo Scientific, Rockford, IL, USA) and developed on X-ray sensitive film.

### Quantification of differentiation marker expression in 3D cultures

3D culture differentiation analysis was performed by quantifying the total expression of the differentiation marker proteins and by measuring the thickness variation of the cornified layers. Pictures were taken with the same objective and camera exposure parameters and processed using Image J 1.04 software. The percentage of K1 or FLG positive area were quantified using thresholds set on the control images.

## Results

### DNA damage and repair in 2D N/TERT-1 keratinocytes

**Figure 2:**
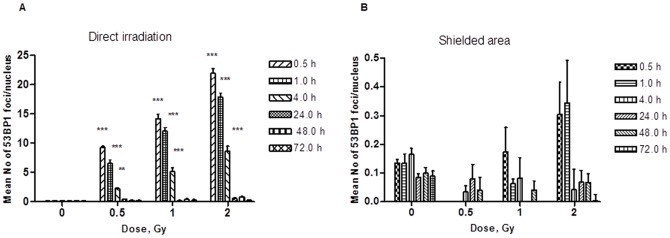
53BP1 foci induction in 2 D N/TERT-1 keratinocytes cultures. Directly irradiated (A) and under the shielding ≈2.2 mm away from irradiation (B) *p<0.05, **p<0.01, ***p<0.001 indicate significant statistical difference to background; Two-way ANOVA analysis, Bonferoni post test.

Irradiation of N/TERT-1 keratinocytes in 2D culture induced linear dose dependent formation of 53BP1 foci with a peak at 30 min post irradiation. The average number of foci per cell was 14.2±0.6 foci/Gy (Figure 2A), which is consistent with previously reported numbers of 15 foci/Gy/cell [31]. Interestingly for the experiments where half of the samples were shielded, the number of foci per cell detected in the shielded part of the samples (>2.2 mm away from irradiation) also showed evidence of DNA damage up to 4.3 times higher than background (0.64 foci/Gy in comparison to 0.14 foci/cell in the control samples at 30 min post 1 Gy) (Figure 2B). Results statistically significant at 0.5–1 h for 1 and 0.5–4 h for 2 Gy.

Disappearance of 53BP1 foci indicates a repair kinetics following a biphasic exponential decay (Table 1). The fast component of the repair is believed to be responsible for 80–90% of the DNA DSB repair after X-ray exposure [32]. In our experiments, the 53BP1 foci seemed also to be mainly repaired by the fast repair component (50% repaired after 11.7 min) although data suggest an increase of the percentage of the slow component with increasing the dose (Table 1). Despite this, N/TERT-1 cells in 2D cultures were successfully able to repair the radiation-induced damage as the number of foci/cell was back to control levels within 24 h for the directly exposed cells and 72 h for the bystander ones (Figure S1 in File S1).

**Table 1:** Two phase exponential repair of 53BP1 foci in 2D N/TERT-1 keratinocytes.

	Fast repair parameters			Slow repair parameters	
Dose [Gy]	Initial damage (d) [foci/nucleus]	b [min]	Fast repaired damage (a) [%]	c [min]	Slow repaired damage (1-a) [%]
0.5	13.6±0.3	4.8±0.6	81.4	59±3	18.6
1	16.7±0.7	11±8	45.7	29±20	54.3
2	28±2	8±3	51.3	57±23	48.6

Slow and fast repair parameters^*^.

* *values±Standard Error; where*

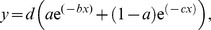

*y = average number of foci per cell, d = total initial number of foci induced by radiation (dose dependent parameter), a = fraction of fast repaired damage, (1-a) = fraction of slow repaired damage, b = rate constant for the fast repair, c = rate constant for the slow repair*.

The percentage of the slow and the fast damage repair component were calculated using the equation from [32]:
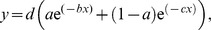
y = average number of foci per cell

d = total initial number of foci induced by radiation (dose dependent parameter)

a = fraction of fast repaired damage

(1-a) = fraction of slow repaired damage

b = rate constant for the fast repair

c = rate constant for the slow repair

### DNA damage and repair in 3D organotypic skin culture

**Figure 3:**
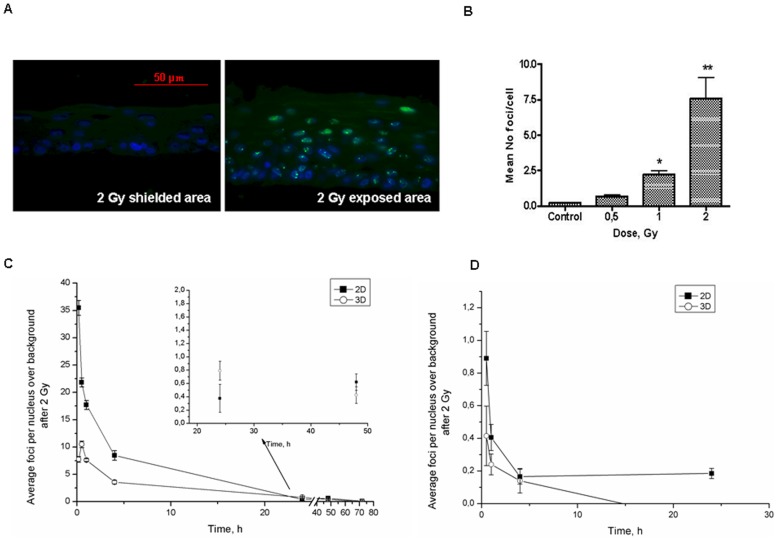
53BP1 foci 30/TERT-1 3D organotypic skin cultures. A – typical examples of foci, B – dose-dependance in foci induction; statistical analysis (n = 3) t-test: *p<0.05; **p<0.01 difference to background level. Panel C, D show 53BP1 repair kinetics of 2D cultures Vs 3D model for both direct (C) and bystander effect (D). Green staining- 53BP1, blue – DAPI nuclear staining.

For the 3D cultures, DNA damage foci after direct irradiation have been scored only in the basal layer (Figure 3). It has been previously reported that 100% of the basal cells were DNA damage foci positive after irradiation [18] with the number of foci/cell decreasing towards the cornified layer as the cornified cells are usually fully differentiated, non-dividing and foci free. The basal layer provides a niche for actively replicating stem cells in the epidermis and that is the reason for its high sensitivity. In the upper layers due to the terminal differentiation process cells have switched off the ability to replicate and, towards the cornified layer, gradually lose their nuclei. At 30 minutes post irradiation, we observe clear dose-dependent increase in the number of 53BP1 foci (Figure 3). However, the average number of foci/Gy/cell was significantly lower (3.8±0.28 foci/Gy/cell) compared to the 2D model. Similar observations after low LET exposures have previously been reported [17] (4.01 foci per cell per Gy in organotypic skin cultures). This result was attributed to the low number of cells in the 3D section scored and difficulties in distinguishing between the foci overlaps especially with increasing the dose. Our data were obtained from three independent experiments with two replicate slides per experiment, each containing at least two different sections and >50 basal cells scored per section. Particular care was taken in scoring foci throughout the whole cell nucleus by moving the objective focal plane through its entire depth. Due to the relatively low number of foci/cell observed, significant foci overlap can also be excluded from our studies. Additional experiment to test if the presence of supporting collagen I, the feeder fibroblast cells or the processing in paraffin could be possible reason for the decreased number of foci in 3D has been performed. N/TERT-1 cells were plated on J2-containing collagen I gels and 18 h after attaching have been irradiated with 2 Gy X-rays or sham irradiated, The cells were still in monolayer at that time. They were fixed 4 hrs post irradiation and processed the same way as 3D cultures. The results showed comparable with the 2D cells on plastic 7.3±0.49 foci per cell (8.6±0.87 in cells on plastic) (Figure S2 in File S1). Control had higher but not statistically significant number foci on the collagen gels and shielded area had comparable number (Figure S2 in File S1, inset table).

The 53BP1 foci level peaks at 30 min post 2 Gy irradiation in 3D cultures while the maximum in 2D was observed at 10 min post irradiation. Moreover, we have observed substantial differences between 2D and 3D repair kinetics with 53BP1 foci disappearing at a significantly slower rate in 3D and persisting up to 24 h post-irradiation (Figure 3C) for the direct effect. Differences in the foci size were also observed with an average of the FWHM of 0.59±0.05 µm for the 2D cultures compared to 0.68±0.05 µm for the 3D models (Figure S3 in File S1).

### 53BP1 foci induction after Gallium Microcollimator irradiation – direct and bystander effect

DNA damage in targeted and bystander cells was also assessed using the Leman Microcollimator and exposing the 3D cultures to 1 µm wide stripes of 30 kVp X-rays at a dose rate of ∼0.014 Gy/min. Due to the minimum distance at which the samples could be placed relatively to the microcollimator (17 mm), the dose spread across a strip with FWHM ∼130 µm (Figure 1B). The distance between the lines was ∼350 µm. A minimum of 50 cells from two samples in each experiment were scored in the central part of the lines (dose >65% of peak) and between the irradiation lines for the bystander experiments (where scattered dose was negligible).

**Figure 4:**
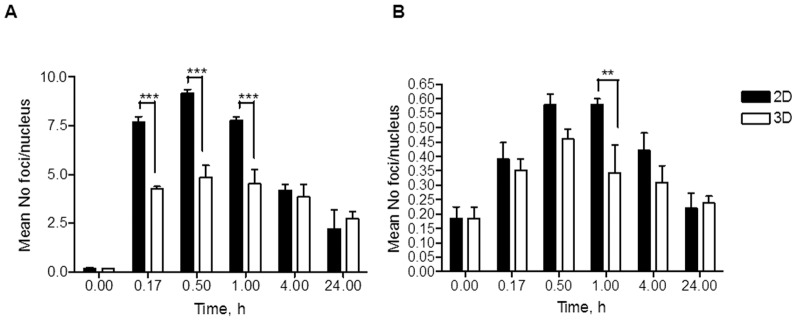
53BP1 foci induction and repair in 2D N/TERT-1 keratinocytes and N/TERT-1 derived 3D skin cultures. Foci in directly irradiated (A) and bystander (B) cells after 30 kVp X-rays micro-irradiation (1 µm slits, 8 min exposure, dose ∼0.095 Gy, (n = 2)). **-p<0.1; ***-p<0.001; t-test.

The peak in foci induction in 2D conditions was at 30 min post-irradiation (30 kVp) for both directly irradiated and bystander cells, with 9.18±0.18 and 0.58±0.04 foci/cell respectively for 0.095 Gy (Figure 4A and B). For the 3D cultures, the foci induction was also peaked at 30 min with 4.9±0.6 foci/cell for direct irradiation and 0.46±0.04 foci/cell for the bystander cells for 0.095 Gy (Figure 4A and B). These foci levels were substantially higher (factor ∼10) than the extrapolated values from the 225 kVp broad field experiments. Statistically significant differences in the number of foci between the 2D and 3D conditions were only observed for the directly irradiated samples. As for the 225 kVp broad field exposures, repair kinetics in 3D appears again to be slower than in the 2D cultures. Interesting the average number of foci detected in the bystander cells (both in the 2D and 3D cultures) appear to be always the same independent of the irradiation setup (i.e. 225 kVp shielding and 30 kVp microcollimator). Considering the lack of scattered dose received by the unexposed cells when using the microcollimator setup, these data indicate DNA damage being induced away from the directly exposed areas by bystander signalling.

In order to evaluate if the X-rays energy (30 kVp) used in the microcollimator experiment is responsible for the increase in the foci yield per Gy compared to 225 keVp irradiations, we additionally performed an experiment in which the 2D cells were irradiated with broad field 30 kVp X-rays. Results showed a 2.7 to 3.8 fold increase in foci induction compared to the factor of 10 experimentally measured (Figure S4 in File S1). Moreover, the 30 kVp broad field irradiated samples had no residual foci at 24 h post irradiation whilst evidence of DNA damage was still present in the microcollimator irradiated cells These results highlight the significant difference in response of both 2D and 3D cultures to localized radiation exposures.

### p21 expression in 2D N/TERT-1 keratinocytes and 3D skin cultures

**Figure 5:**
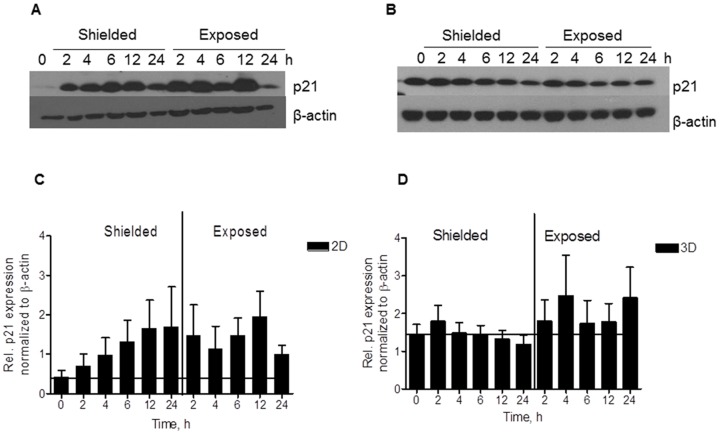
p21 expression after 2 Gy irradiation. Expression in N/TERT-1 keratinocytes in 2D (A) and 3D conditions (B). Densitometry results from two independent experiments in replicates (C and D).

p21 CDK inhibitor has been described as a factor involved in the G1 cell cycle arrest post irradiation [5]. The levels of p21 regulate via a feedback loop the phosphorylation of p53 upstream and the increase of p21 is connected to subsequent apoptosis or induction of terminal differentiation reported in skin [4]. In both the directly irradiated and shielded 2D cultures, the p21 expression shows time-dependent induction post 2 Gy irradiation and very low basal levels (Figure 5). We also showed concomitant increase in p21 levels in the 2D cells with the increase of DNA damage foci (Figure S5 in File S1) proving that p21 is one of the factors activated form the DNA damage itself. p21 induction follows similar trend in the irradiated and the neighboring areas as the peak is reached about 12 h after the exposure and then the levels decrease considerably at 24 h. For the 3D cultures, the basal CDK inhibitor levels are higher than in 2D monolayer cells. There are also reports that in differentiation skin tissues the basal p21 levels are high [5]. In this case, the induction after irradiation is not statistically significant and it remains at basal levels.

**Figure 6:**
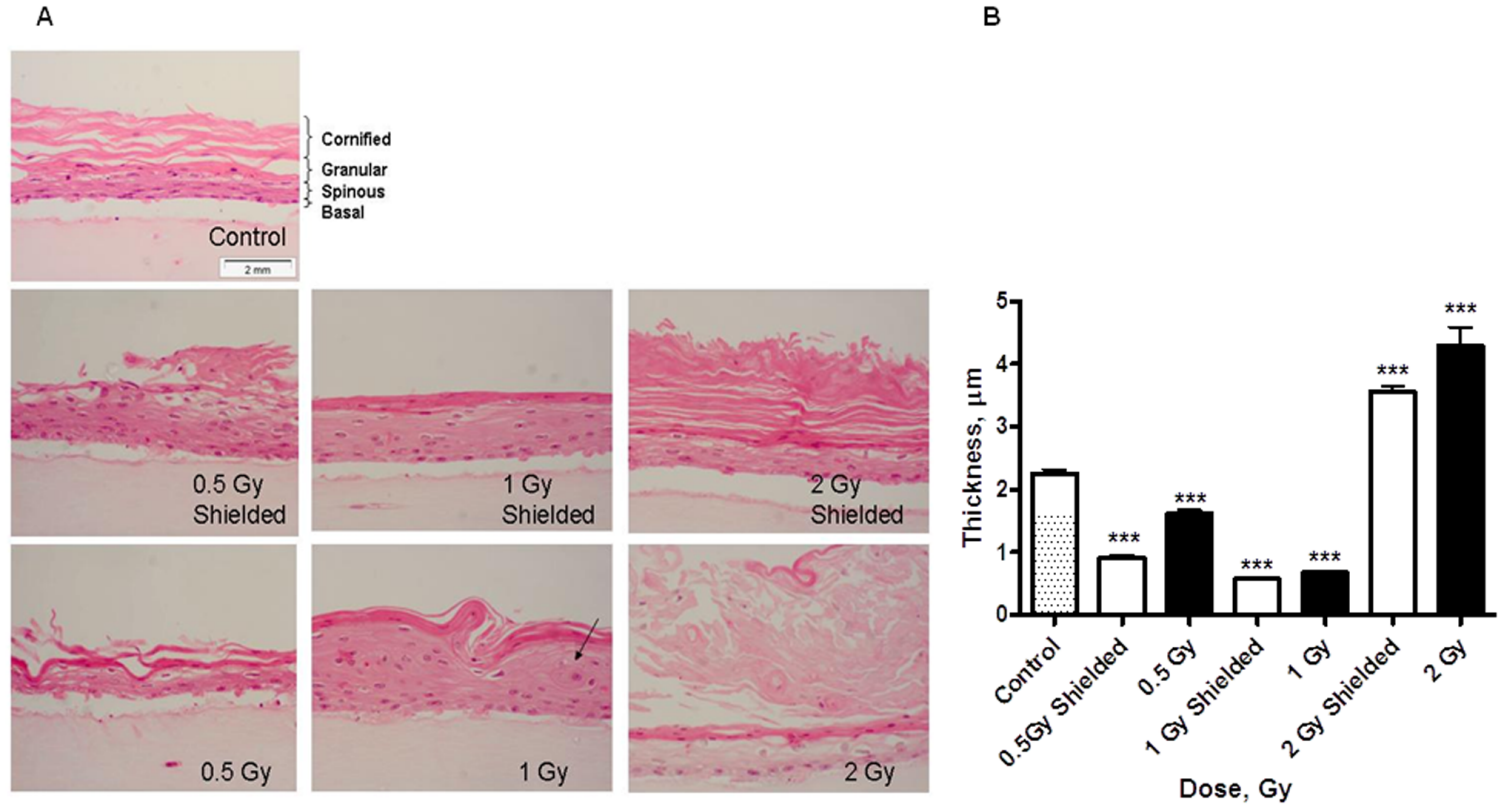
Morphological analysis of half shielded 3D organotypic skin cultures 7 days post irradiation. H&E staining (A). Quantification of the cornified layer thickness of the H&E stained samples (B). * - p<0.05; **- p<0.01; ***- p<0.001, One-way ANOVA analysis, Tukey post-test.

The hematoxylin and eosin (H&E) staining of N/TERT-1 organotypic skin cultures showed clear morphological changes after irradiation. The thickness of the cornified layer has been assessed in ten different areas from five visual fields per each 3D culture using calibration from Image J software. In total the average thickness of two 3D cultures per experiment from two independent experiments was measured. Apoptotic foci were visible in the granular layer (arrow) and the cornified layer exhibited inflammatory-like phenotype (Figure 6A). At the higher dose points (2 Gy), the cornified layer in both the exposed and shielded part of the samples was hyperproliferative and statistically significant thicker than normal cultures (Figure 6B) while lower doses (<1 Gy) seems to have the opposite effect. This is consistent with an inflammation response that has a threshold of activation, with gradual accumulation of pro-inflammatory signals. The signals appear to be transmitted also to the non-irradiated areas.

**Figure 7:**
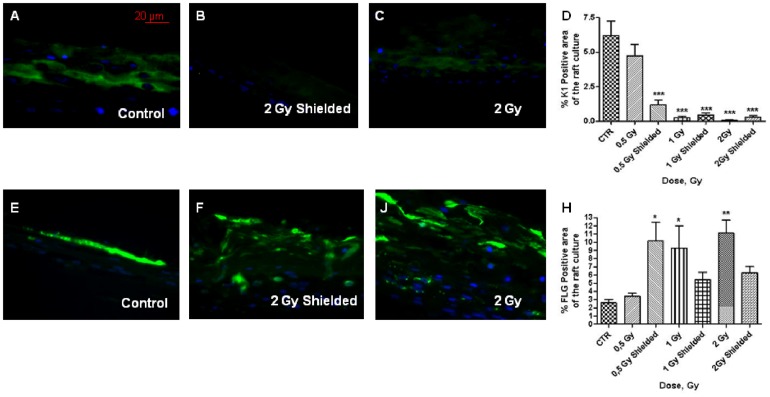
Cytokeratin 1 and filaggrin expression in half-shielded 3D cultures 7 days post irradiation. Cytokeratin 1 expression (A–D) and filaggrin expression (E–H). Results (D and H) are mean from 2 independent experiments, 2 replicate slides per experiment and 5 visual fields with 145 µm length per each slide;*** - p<0.001, One way ANOVA analysis, Tukey post-test. Scale bar 20 µm.

To further investigate this, we monitored expression of cytokeratin 1 (K1), an early differentiation marker. K1 levels have been tested after irradiation in 2D keratinocytes as there were not observed changes in the levels in monolayer cells (Figure S6 in File S1). For comparison the monolayer cells were treated with 2.8 mM CaCl_2_ used as a positive control, since Ca^++^ is one of the early signals for keratinocyte differentiation and induce K1 overexpression [33]. Our data indicate a fast dose-dependent decrease in the K1 expression in both directly irradiated and shielded areas in 3D cells (Figure 7A, B, C and D). During the normal process of terminal differentiation K1 expression is increasing. Decrease of this early marker has been observed during re-epithelization and wound healing [34] where hyper proliferation is also occurring. Finally, we also monitored Filaggrin (FLG) expression. FLG has very important role during the terminal differentiation of keratinocytes as it cross-links the keratin filaments. It is a late differentiation marker which is monitored for normality and completeness of the differentiation process. Statistically significant higher levels of Filaggrin positive staining cells were found within the suparabasal layers of the directly exposed 3D cultures in dose dependent fashion (Figure 7H). Increase in filaggrin expression was also detected in the shielded areas. The highly increased expression in the outer layers of the 3D model is a marker for premature terminal differentiation [34]. The data suggest an significant overall increase in the process of terminal differentiation that affects not only the directly irradiated but also the shielded areas (distances up to 9 mm from the irradiated regions).

### Cell signaling in directly exposed and shielded areas of 3D N/TERT-1 skin cultures

**Figure 8:**
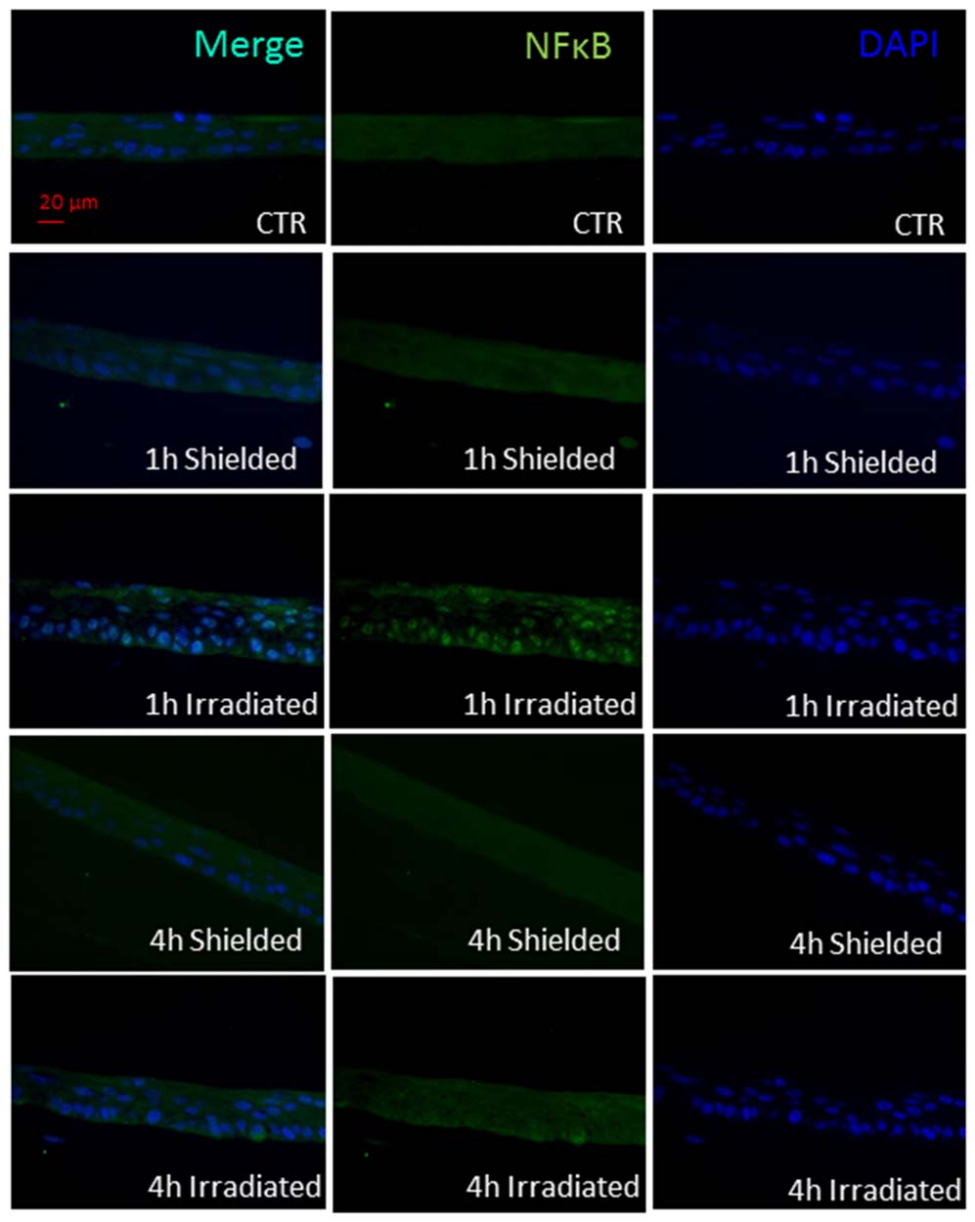
NF-κB phospho-p65 nuclear translocation. Pannels show localization of the p-p65 unit 1 and 4 h after half-shielded 2 Gy irradiation of 3D skin model. Green – p-p65.

As an initial signal from the DNA damage we monitored the nuclear translocation of NF-κB transcription factor (Figure 8). In our 3D culture system this re-localization occurred as early as 1 h post irradiation (Figure 8). The levels of NF-κB decreased at 4 h post exposure.

**Figure 9:**
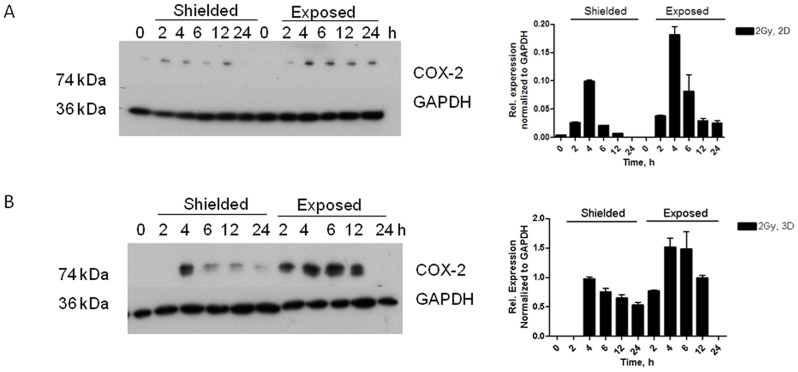
COX-2 expression in 2D N/TERT-1 cells and 3D organotypic cultures. COX-2 induction was observed in both irradiated and shielded areas of the 2D and 3D cultures and confirmed by quantification of the relative COX-2 expression (A, C). Dose-dependent induction of COX-2 in 3D skin cultures (B) Graphs represent densitometry analysis of of A, B and C respectively and are the mean of two independent experiments in replicates.

Western blotting for the downstream target of NF-κB, COX-2 has showed up-regulation in both exposed and shielded areas in both 2D cells (Figure 9A) and 3D cultures (Figure 9C) with peak at 4–6 h post irradiation and increasing with dose (Figure 9B). These observations were connected with statistically significant increased levels of the COX-2 enzyme product PGE_2_ in culture media 72 h post irradiation (Figure S8B in File S1).

**Figure 10:**
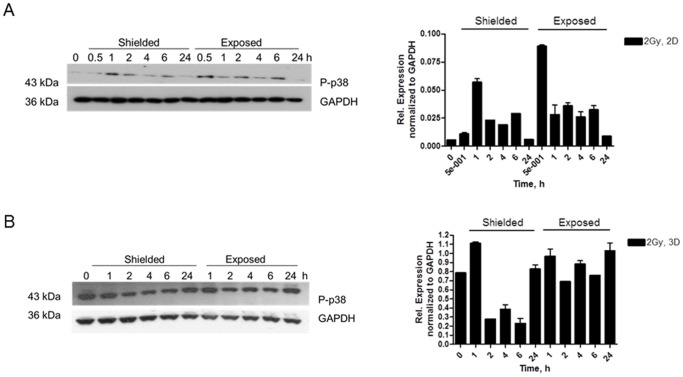
p-p38 expression in half-shielded 2D (A) and 3D (B) N/TERT-1 keratinocyte cultures after 2 Gy irradiation. (B) Quantification of the p-p38 relative levels, normalized to GAPDH in 2D (B) and 3D N/TERT-1 cell cultures (D). Results mean from two independent experiments in replicates. SEM-standard error bars. *-p<0.05, One-way ANOVA, Tukey post test.

According to the literature, p-p38 is one of the COX-2 translational activators [35]. p38 MAPK is activated from extracellular stimuli as stress and cytokines, including ionizing radiation and TNF-α [3] and covers important roles in the cell differentiation, growth inhibition and apoptosis. Consequently, p38 has been classified as a tumour suppressor [36]. In our model p-p38 induction was observed as early as 0.5 hrs in partially exposed to 2 Gy monolayer of N/TERT-1 cells (Figure 10A) with up-regulation detected up to 6 h post irradiation (Figure 10A). Using the same experimental set up, no statistically significant variations were observed in the p-p38 levels in the 3D cultures (Figure 10B). This could be due to the ambiguous role of p38 as driving factor for induction of the differentiation marker involucrin and promotion of the keratinocyte apoptosis [37].

## Discussion

### DNA damage and repair in 2D and 3D models

Significant differences were observed between the 53BP1 foci induction in 2D and 3D directly irradiated cultures. Studies reporting similar findings have indicated foci overlap, the limited number of cells/section and inhomogeneity in dose distribution as a possible cause of the low number of foci detected in 3D samples. This is unlikely in our study as a significant number of cells were scored and at the doses used, foci were quite sparse and with negligible chance of overlapping. Moreover, foci were scored using a high magnification objective (×63) and by moving the focal plane through the whole cell nucleus thickness to assure a comprehensive foci account. Finally, the high energy X-ray used (225 kVp) and the sample limited thickness (∼200–250 µm) result in a very homogeneous dose distribution across all layers of the exposed construct. The difference in the number of DNA damage foci detected in 2D and 3D must therefore be due to the intracellular signalling which regulates cell activities for the differentiation processes in the 3D constructs. The fraction of residual DNA damage (24 h) is also higher in the 3D model implicating a slower repair mechanism in 3D as highlighted by the repair kinetics data. These data could be explained considering that speed and efficacy of DNA repair strongly depends on the chromatin organization and how accessible the DNA is to the repair machinery. In the basal layer of the 3D skin model there are always cells entering the terminal differentiation process and therefore stopping proliferation to enter a quiescent mode. The terminal differentiation of keratinocytes has also been regarded as a type of apoptotic process and has been reported to use the same cellular machinery [22]. Quiescent, differentiating cells have more compact chromatin organisation making them more refractory to 53BP1 foci formation [38] but also resulting in a substantially slower DNA repair [39]. This would account for the lower number of 53BP1 foci detected in the 3D cultures and their persistence up to 48 hrs.

p21 has a role of universal inhibitor of cyclin-dependent kinases (CDKs) inducing G1/S arrest by binding and blocking the phosphorylation of the retinoblastoma protein. p21 has been shown to perform opposing roles – inducing or preventing apoptosis and inhibiting or promoting differentiation [4]. Its increase has been suggested as a necessary step in the removal of cells with accumulated DNA damage via apoptosis and responsible for cell cycle arrest to facilitate repair of sub-lethal DNA damage [4]. The protein was also reported to be up-regulated in the initial phases of human primary keratinocyte terminal differentiation but to decrease at the late stages in order to promote terminal differentiation [5]. Evidences of dose-dependent induction of p21 1–4 h post X-ray irradiation have been previously reported for thyroid cells [4]. In irradiated 2D monolayer keratinocytes, we also observed p21 induction combined to significant decrease of actively proliferating cells (Figure S7 in File S1). p21 levels return to background level 24 h post irradiation. This could be connected to removal of the DNA damage containing cells through apoptosis and leaving only the successfully repaired population as the whole process is completed within 24 h. However, the organotypic 3D cultures exhibit a different p21 expression pattern. They have high basal levels of p21 which remained constant following irradiation and only at later time gradually decreased. In the 3D model, the high p21 basal level is connected with on-going cell differentiation [5]. In comparison, in 2D cells grown on monolayer and exposed to 2 Gy irradiation 18 h after plating with detection of p21 levels 12 h later, only the presence of collagen I in the system was not able to trigger increased p21 basal levels (Figure S9 in File S1). These findings highlight a key dual role of p21 in the DNA damage response: triggering apoptosis in 2D conditions while stimulating terminal differentiation in 3D environment. Both can be regarded as protective mechanisms aimed at preventing cells with unrepaired DNA damage progressing through the cell cycle and dividing. According to such hypothesis, terminal differentiation (when possible, i.e. in 3D tissue models) is preferred to induction of apoptosis. However, the situation might be even more complicated as the effect at lower doses might be dependent on reaching of certain cytokine concentration to trigger the morphological changes (Figure 6). This could lead to the thinner cornified layer at lower doses, when apoptosis would be main way of cell removal and the tissue could deal with resorbing the dead cells.

### Bystander effects in 3D cultures

The foci induction in half-shielded experiments showed dose dependent induction of non-targeted DNA damage as early as 10 min post irradiation in both 2D and 3D samples. Although half-shielded samples also receive some scattered dose (estimated to be around 2% of the directly exposed cells as by Gaffchromic film measurement), this alone cannot account for the DNA damage detected in the shielded cells. Microcollimator experiments (where the scattered dose to shielded samples is reduced to negligible levels) also result with significant number of 53BP1 foci in cells between the irradiated lines. These, however, appear to be dose independent suggesting that both direct and bystander damage coexist at very low doses. Considering the level of residual DNA damage at 24 h in irradiated and shielded cells, we can also conclude that bystander damage is repaired more efficiently than direct damage suggesting possible implication of different mechanisms. More interestingly, shielded areas of the exposed 3D cultures also show evidence of altered differentiation with matching similarity to the differentiation changes observed in the directly exposed part of the samples. The changes observed in our 3D model indicate that an abnormal phenotype of the skin cultures regarded as a precursor of the late radiation effects can be induced in both the directly exposed and in the shielded areas of the samples. The fact that the effect was transmitted to the non-irradiated neighbouring areas and the presence of bystander DNA damage in these areas suggest that there is combined action of soluble pro-inflammatory factors and damaging molecules such as ROS. However, the DNA damage foci in the bystander areas are completely repaired 24 h after irradiation but despite this, shielded regions still develop an inflammatory phenotype 7 days post irradiation, implicating that the presence of long-term DNA damage might not be necessary for development of the morphological abnormalities. The presence of cells with elevated DNA damage foci have previously been reported in chronic inflammatory conditions accompanied by high levels of cytokine secretion [39]. A plausible hypothesis, in our model, is that directly irradiated cells with persistent foci are responsible for the secretion of pro-inflammatory factors that could therefore cause DNA damage and morphological changes away from the irradiated area.

### Mechanisms of cell signaling activated in 3D skin cultures post localized irradiation

**Figure 11:**
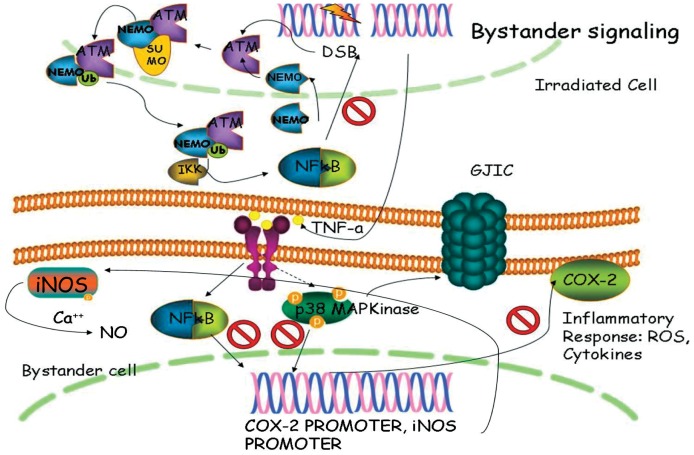
Scheme of the proposed radiation-induced signalling in directly irradiated and bystander cells in 3D organotypic skin cultures. NF-κB is activated in the directly targeted cells from the DNA damage. It leads to secretion of TNF-α which binds to receptors in non-targeted cells and activates the intracellular NF-κB and p38 MAPK. Cx43 GJIC is also feasibly involved in the bystander signal transmission. Active NF-κB and p-p38 activate COX-2 expression and the product of COX-2 PGE2 is responsible for amplification of the pro-inflammatory signaling throughout the tissue. The red symbols show possible target points for inhibition in order to control the signaling.

Cell signaling in skin after an external insult is a complex network with numerous key-player enzymes, transcription factors and signaling molecules [40]. The target genes (including COX-2, iNOS, genes coding the cytokines and chemokines (IL-1; TNF-α; TGF-β; IL-8 etc.) are responsible for production of secondary signals that propagate to the neighbouring cells. This whole cascade has been suggested, in different publications, to have a role in the development of the early and late skin radiation-induced effects [39]–[42]. We also explored some of these cytokines release from the 3D cultures post irradiation (Figure S8 in File S1). There was indication of increase in TNF-α and statistically significant increase in the enzyme product of COX-2 PGE_2_. Based on the results from the current work we suggested the mechanism described on Figure 11 as the signaling pathway operating between the directly irradiated and non-irradiated cells in human epidermis post irradiation that is responsible for the development of DNA damage and pro-inflammatory responses in the bystander regions.

Our experimental work revealed that NF-κB pro-inflammatory and apoptosis regulating transcription factor is one of the early activated molecules in the radiation-induced signaling cascade in 3D skin model. The effect of NF-κB phosphorylation and nuclear translocation is visible as early as 1 h post irradiation in the directly irradiated cells (Figure 8). This immediate response could be explained by the ability of NF-κB to be activated directly from the presence of DSB in the cells [39]. In our model this activation and nuclear translocation of NF-κB was observed only in the directly irradiated areas of the 3D cultures. However, late effects of inflammatory-type reactions and abnormal differentiation were also observed in the neighboring areas of the 3D skin. Soluble signals transmitted via paracrine mechanisms towards the non-exposed parts of the cultures could be responsible for such effects [43]–[44]. COX-2 has been described over the last 10 years as one of the pro-inflammatory enzymes responsible for the radiation-induced bystander responses [45]–[46]. We found both dose and time-dependence for COX-2 induction in our skin model. The p38 MAPK pathway is shown to be directly activated by IR in directly irradiated and also in bystander cells [36], [46]–[47]. Early formation of active p-p38 1 h post irradiation has been observed in both irradiated and shielded parts of the 3D culture, suggesting again that soluble molecules (diffused or transported from the site of the initial DNA damage) have been involved in its activation. A further connection between p38 MAPK signaling as an intermediate enhancer of the COX-2 activation is via NF-κB and this is also very likely to be involved in our model system. Accordingly a plausible hypothesis supported by our data, firstly foresees activation of NF-κB, then early increase in the intercellular signal transducing p38 MAPK at 1–2 post irradiation and finally activation of the secondary signaling molecules producer – COX-2 in a possible sequence NF-κB→pp38→COX-2. This suggestion has been based on our data and data from [35] showing connections between DNA damage and the three molecules in the conditions of cellular endoplasmic reticulum stress. Our data support the hypothesis that COX-2 is likely to be responsible for a long range, late effects in both directly irradiated and bystander areas at 3D tissue level, which makes it a suitable target for reduction of the radiation-induced inflammatory reactions in skin. Further studies focused on blocking the steps in the cascade will be necessary to confirm the sequence of the implicated signaling cascade.

## Supporting Information

File S1 **Supplementary Figures S1–9.**
(PDF)
