# Supplementary material for: Mechanisms of DNA Damage Response to Targeted Irradiation in Organotypic 3D Skin Cultures
Source: PLoS One. 2014 Feb 5;9(2):e86092. doi: 10.1371/journal.pone.0086092 (PMC3914781; doi:10.1371/journal.pone.0086092)
Supplement: File S1 — Supplementary Figures S1–9. (PDF) [file pone.0086092.s001.pdf]

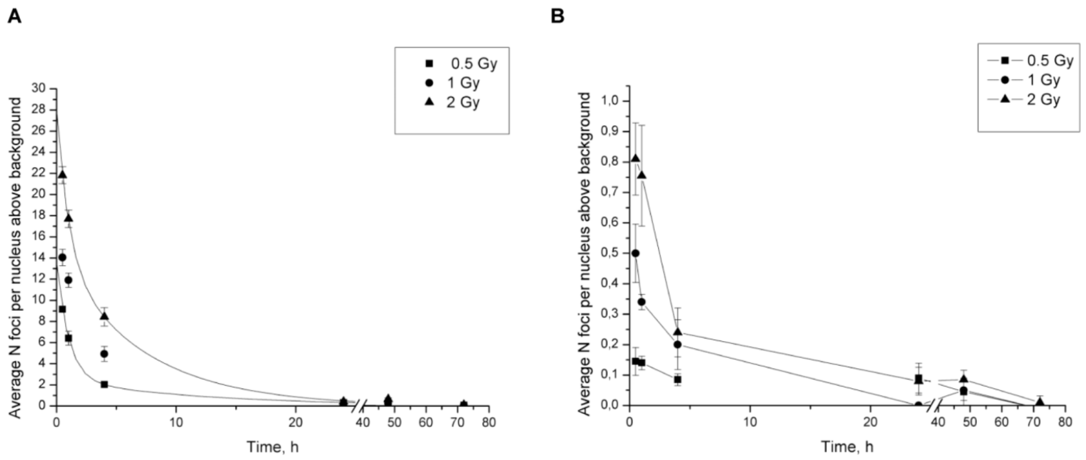

Figure S1. Repair kinetics of 53BP1 foci in 2D N/TERT-1 keratinocytes – direct (A) and bystander (B) effects. (A) Two-phase exponential decay fitted 53BP1 foci repair curves in 2D N/TERT-1 keratinocytes direct irradiation (n=3). Data fitted with Origin 8.0 Software (OriginLab Corporation, Northampton, MA, USA). Error bars represent the SEM. (B) Point-to-point 53BP1 foci repair curves in 2D N/TERT-1 bystander cells (n=3). Results not statistically significant different (One-way ANOVA, Tukey post test; p>0.05). Error bars represent the SEM.

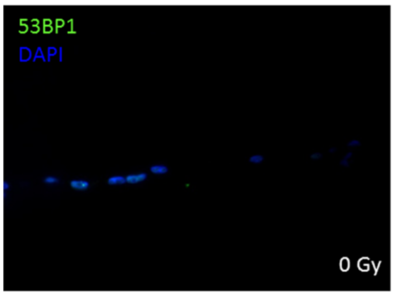

	0 Gy	2 Gy	2 Gy Sh
2D N/TERT-1	0.17±0.02	8.6±0.87	0.41±0.08
2D N/TERT-1 on collagen gels	0.48±0.13	7.3±0.5	0.64±0.17

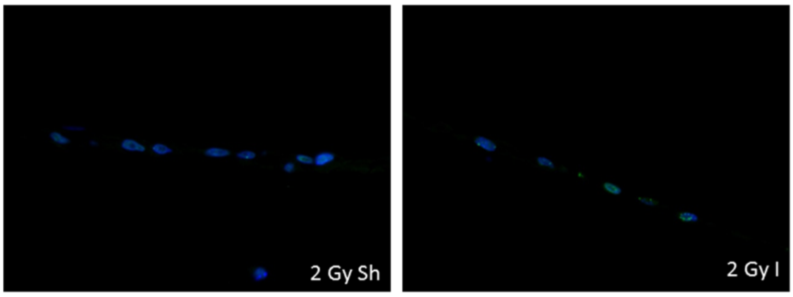

Figure S2. 53BP1 foci formation in 2D N/TERT-1 cells plated on J2-3T3 fibroblasts containing Collagen I gels. The cells have been plated on the gels, let to attach for 18 hrs and irradiated with the same set-up as the 2D cells on plastic dishes. Fixation and processing for immunofluorescence were the same as for the 3D cultures. The data in the inset table are

for 4hrs post irradiation obtained from 2 independent experiments in replicates. No significant differences within the foci induction in both 2D conditions. Sh – shielded, I – irradiated.

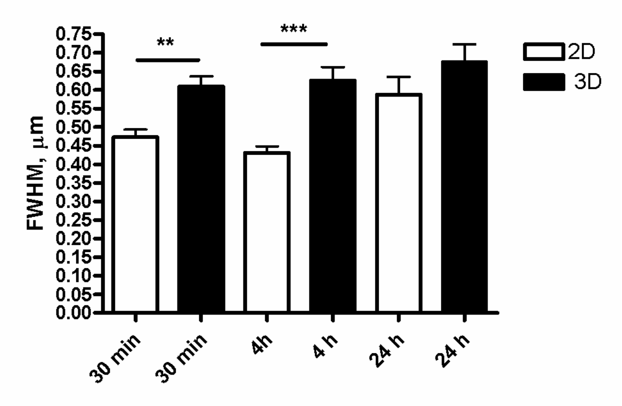

Figure S3. 53BP1 foci size in 2D keratinocytes vs. 3D organotypic skin cultures after 2 Gy irradiation (n=2). Statistical analysis – One-way ANOVA, Tukey post test; **$p<0.01$, ***$p<0.001$. Error bars represent the SEM.

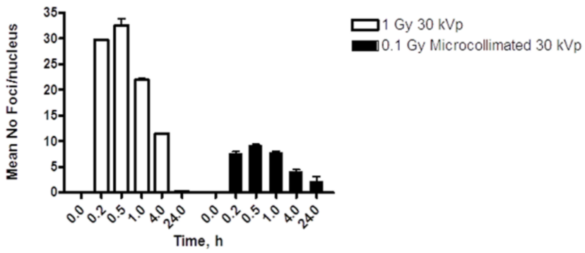

Figure S4. Comparison between 53BP1 foci formation in 2 D cells after 1 Gy 30 kVp broadfield X-rays and 0.1 Gy microcollimated 30 kVp X-rays. The factor between the foci formation number in the 2 set ups is only 3 and the dose factor – 10. This mean that there are ~3.33 times more foci formation after microcollimated irradiation that it has been extrapolated from the broadfield exposure (n=2).

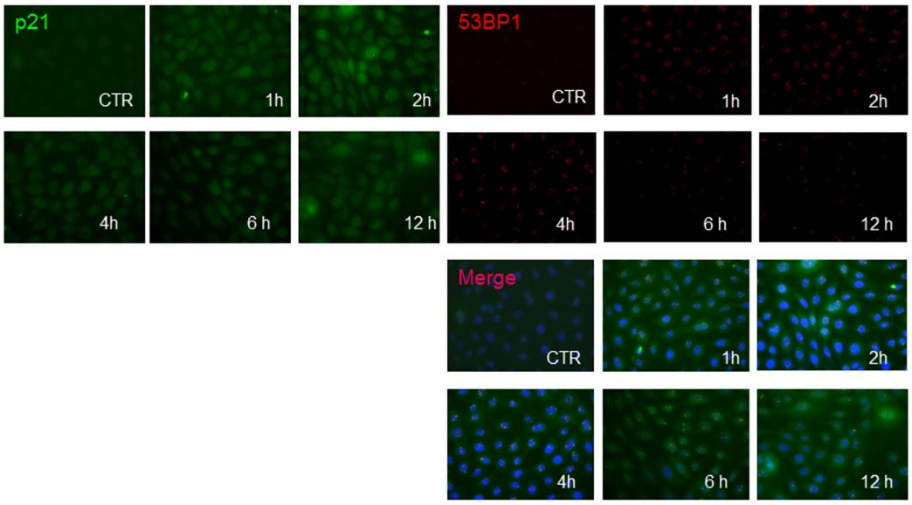

Figure S5. Co-induction of p21 and 53BP1 DNA damage foci in 2D N/TERT-1 cells after 2 Gy 225kVp X-rays. The concomitant induction of p21 and 53BP1 was maximal at 2 hrs post irradiation suggesting DNA-damage connected p21 activation. While foci were disappearing at 12 hrs post irradiation, p21 was still detectable. Blue-DAPI, green-p21, red-53BP1.

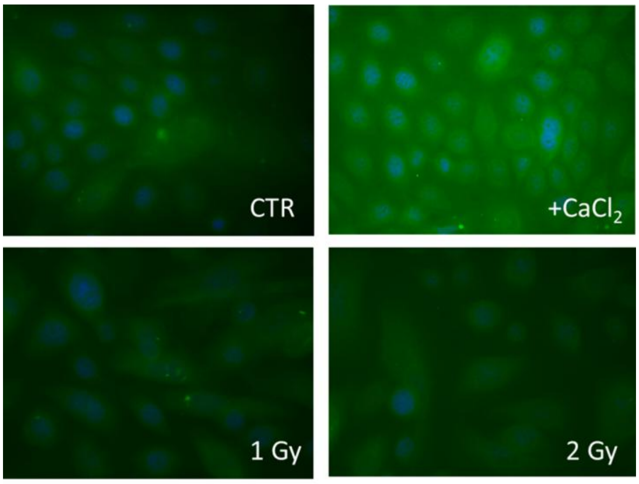

Figure S6. K1 (early differentiation marker) expression in 2D N/TERT-1 72 hrs post irradiation. $CaCl_2$ (2.8 mM) was used as positive control inducing K1 expression. No K1 induction in 2D cells after irradiation. Blue-DAPI, green-K1.

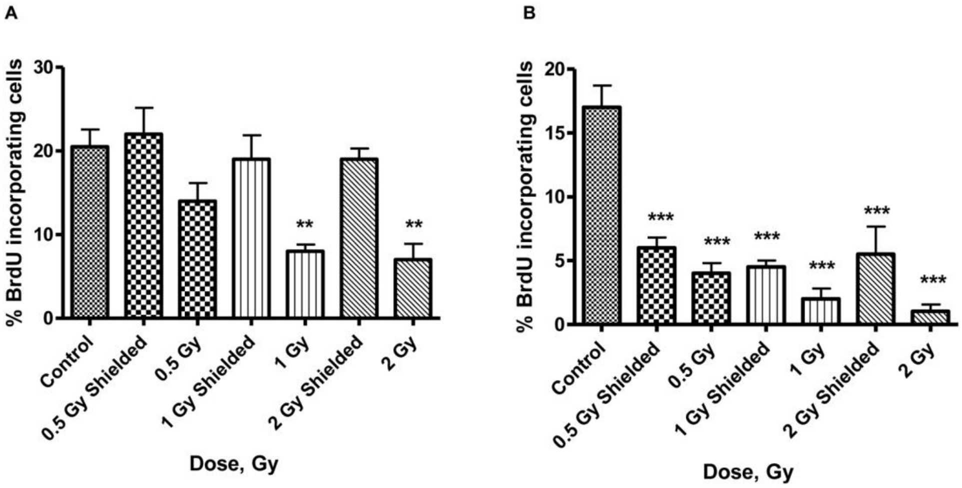

Figure S7. Proliferation assay – BrdU incorporation. (A) Half-shielded monolayers of N/TERT-1 keratinocytes 24 h post irradiation (n=2). (B) Quantification of % BrdU incorporating basal cells of 3D skin cultures 7 days post irradiation. Results from (A) at confluence and (B) at fully differentiated state, cells have been pulsed overnight with BrdU for both conditions. Results from two replicate slides per point and 50 cells from replicate. * - p<0.05; **- p<0.01; ***-p<0.001; One-way ANOVA analysis, Tukey post-test.

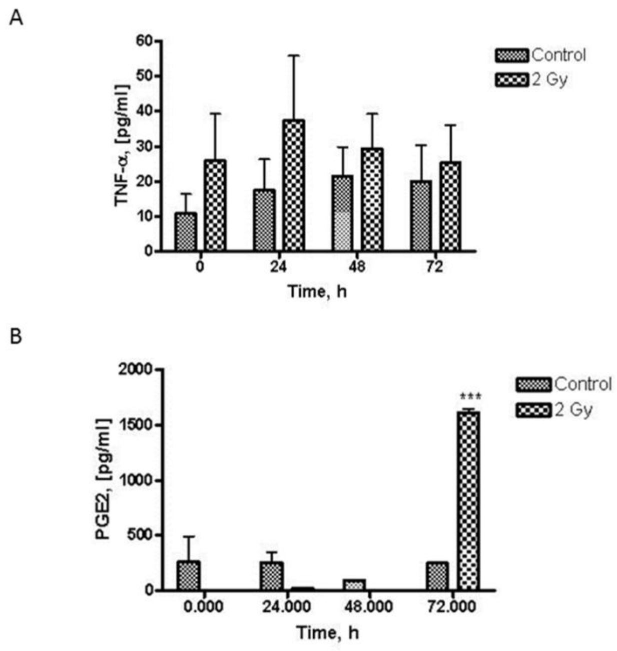

Figure S8. Cytokine secretion in 3D culture media post irradiation. Time dependence of TNF-α (A) and PGE_2 induction in 3D organotypic skin cultures after 2 Gy irradiation (n=3). ***-p<0.001, One-way ANOVA analysis, Tukey post-test.

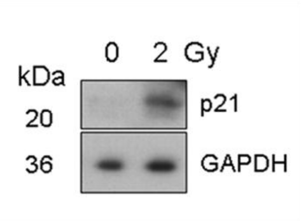

Figure S9. p21 response to radiation in monolayer of N/TERT-1 cells plated on J2-3KT fibroblasts containing Collagen I gels. Cells have been plated on Collagen I, let to attach for 18 hrs and either irradiated or sham irradiated with 2 Gy X-rays. Cells were harvested 12 hrs post irradiation. Results representative from two independent experiments.
